# 

**Published:** 1882-01

**Authors:** 


JANUARY, 1882.—CONTENTS.

PAGE

ORIGINAL COMMUNICATIONS—The Study of Comparative Pathology. By Thomas E. Satterthwaite, M.D. . . . . . 1
The Contagious Diseases of Domestic Animals. By Ezra M. Hunt, M.D. . . . . . 13
Epizootic Suppurative Cellulitis; or, Phlegmonous Erysipelas in Horses. By George H. Berns, D.V.S. . . . . . 29
Expansion and Contraction of the Foot, and How to Shoe it. By John N. Navin, V.S. . . . . . 32
Puerpural Apoplexy. By Thomas Robertson, M.R.C.V.S. . . . . . 43

EDITORIAL—Homœopathy in Veterinary Practice—The Cause of Death in the Colic of Horses—State Veterinary Bureaus—The American Public Health Association and Veterinary Medicine—Do Southern Hogs have Trichinæ?—Acute Jaundice in Sheep—English Veterinary Degrees—Epidemic Catarrhal Influenza—Equine Cemeteries—Discourteous Treatment of an Expert Witness. . . . . 47-54

CASE DEPARTMENT—Mixed Central Sarcoma of the left Humerus with Secondary Deposits in the Lung and Kidneys—Sudor Cruentus or Blood Sweating—Scirrhous Carcinoma of the Bladder—Reticulated Round Cell-Sarcoma of the Orbit, with Secondary Growths Internally containing Melanotic Deposits—Two Cases Diagnosticated as Milk Fever—Abdominal Dropsy Complicating Pregnancy—Colitis; Preceded by Acute Diarrhœa—Acute Articular Rheumatism Secondary to Catarrhal Influenza—Probable Carcinomatous Tumors Encroaching upon the Alimentary Tract—Convulsions in a Bear—Laparo-Hysterotomy . . . . . 55-71

CORRESPONDENCE—Distillery Slops—Spinal Paralysis of the young Carnivora—Thoroughbred 72-73

REVIEWS—Manual of Histology—The Journal of the American Agricultural Association—Books and Pamphlets Received . . . . . 74-76

PROGRESS OF VETERINARY SCIENCE—Rabies, a Possible Cause and Probable Preventive—Precautions against Hydrophobia—Antidote for Hog Cholera—Food for Carrier Pigeons—A Remarkable Mode of Zoölogical Distribution—The Effect of Long continued Minute Doses of Mercury on Animals—Flesh and Fat Producers—Pasteur's Vaccinations—A New Cure for Snake Bites—Controlling Sex—Breeding Mare Mule—Protection of Cattle against Charbon—Hydrophobia and its Cure—The Stomachs of the Horse, Cow, Sheep and Pig—Rapidity of Stomach Absorption in the Dog and Cat—The Digestion of Fishes—Muscular Strength in the Jaw of the Crocodile and Dog—Dog Disease in the Arctic Regions—Diseases Among Texas Cattle—Relative Distribution of Trichinæ Spiralis in the Muscles of the Hog—Extra Uterine Pregnancy in a Quail—The Temperature of Cattle—The Size of the Spleen in Cattle—Action of Light and Darkness, and of Different Colored Lights upon the Respiration of Lower Animals—Chloral v. Strychnia—Contagious Pyæmia of Rabbits—Trichina-like Parasites—A New Mode of Treatment with Chloroform . . . . . 77-88

NEWS AND MISCELLANY—Death of Four Horses from Eating Ensilage—The Number of Cattle in Texas for 1881—Number of Goats in Europe—Tuberculosis—Elephant "Bolivar"—Hog Products—Fasting Dog—M. Richenbach—Three Hundred Horses Burned—Poison in Lupins—Recipe for Sheep Dip Used in Texas—Sheep Scab in Australia—Margosse Oil—M. Pasteur—Thomas C. Cowley—Oil Cake—Consul-General Weaver—White Horses—One Cause of Colic in Horses—A New Veterinary School—An English Buyer of Horses in the United States—Public Health Association—Horses Finding Their Way Home—Consumption of Horse Flesh in Germany—Advance in the Price of Horses—Japanese Food—Influences of Parents—Cattle Restaurants—Claims of Comparative Pathology—Practical Value of Vivisection—A Wonderful Bird—Meat from Cattle Suffering from Splenic Fever—Australian Wild Horses—Exports and Imports from the Port of New York Last Year—The Hippopotamus—Jibbing Horses . . . . . 89-98

SUPPLEMENT TO THE VETERINARY MEDICAL REGISTER. . . . . 99-100

TITLE AND INDEX TO VOLUME II. . . . .

